# Identification of a Male-Specific Amplified Fragment Length Polymorphism (AFLP) and a Sequence Characterized Amplified Region (SCAR) Marker in *Eucommia ulmoides* Oliv.

**DOI:** 10.3390/ijms12010857

**Published:** 2011-01-24

**Authors:** Da-Wei Wang, Yu Li, Zhou-Qi Li

**Affiliations:** College of Forestry, Shaanxi Key Laboratory of Molecular Biology for Agriculture, Northwest A&F University, Yangling, Shaanxi 712100, China; E-Mails: daweiwon@163.com (D.-W.W.); liyu0214@yahoo.cn (Y.L.)

**Keywords:** AFLP, SCAR, male-specific, *Eucommia ulmoides* Oliv

## Abstract

*Eucommia ulmoides* Oliv. is a dioecious species. Currently, there is no method to identify the sex during the juvenile stage that lasts a relatively long time. This study aimed to develop a sex specific Amplified Fragment Length Polymorphism (AFLP) marker for *Eucommia ulmoides* Oliv. Of a total of 64 AFLP primer combinations screened, primer combination E-ACA/M-CTT produced a 350 bp male-specific marker. This 350 bp AFLP marker was converted into a 247 bp Sequence Characterized Amplified Region (SCAR) marker. Results suggest that the SCAR marker can be utilized for early sexual identification in *Eucommia ulmoides* Oliv., and it will greatly facilitate future breeding programs.

## 1. Introduction

*Eucommia ulmoides* Oliv., also called Du-zhong, a dioecious perennial angiosperm, is one of the oldest tonics in Chinese traditional medicine. The dry stem bark of *Eucommia ulmoides* Oliv. was officially listed in the Chinese Pharmacopoeia as an anti-hypertension and bone fracture treatment curative. Recent studies have shown that its bark has many pharmacological actions such as antifungal, inhibiting adipogenic differentiation and antihypertensive, anti-oxidation, anti-inflammatory, and low-density lipoprotein oxidative modification [1–5]. Du-zhong leaves contain many phyto-chemicals, such as polyphenolics, flavonoids and triterpenoids [6]. Du-zhong tea, the aqueous extract of *Eulmoides* leaves, has been already known as a functional food and commonly used for reduction of hypertension [7,8]. Gutta-percha is an industrial gum obtained from the seeds, barks and leaves of *Eucommia ulmoides* Oliv [9,10].

In *Eucommia ulmoides*, male and female trees have different practical uses. Usually the male plants are used for landscaping or as the street trees [11], whereas the leaves of female trees produce high quality vegetable oil that can be made into various health products [12]. The gum content in the female tree is significantly higher than that in the leaf of the male tree, and much higher in the seeds of both than in the bark and leaves [13,14]. In general, female trees are more valuable than the males. Therefore, sex identification in the early stages of growth is of great importance in plant management and utilization.

In practice, the cultivation of the unwanted males leads to wasted resources, which could be avoided if the sex of the plant could be identified at a juvenile stage. The commonly conducted morphological and cytological studies so far have failed to differentiate accurately the sex forms. It takes seven to eight years for the young seedlings to flower in nature, and it is difficult to distinguish the gender by morphological or cytological methods before they flower. Thus, it is necessary to develop a rapid and reliable technique for early sex identification of *Eucommia ulmoides* Oliv.

The AFLP (Amplified Fragment Length Polymorphism) technique was introduced by Vos *et al*. [15]. The major advantages of AFLP are (1) a high multiplex ratio, (2) a limited set of generic primers, and (3) the non-requirement for sequence information. To date, AFLP has been used in combination with BSA (Bulked Segregate Analysis) to identify sex linked markers in several dioecious plants [16], such as *Asparagus officinalis* L., *Dioscorea tokoro*, G*inkgo biloba*, *Pistacia*, *Ficus fulva*, *Rumex nivalis*, *Cannabis sativa* L., *ilex* and *Rumex acetosa* [17–26].

Although the AFLP technique can be used to identify a large number of markers rapidly, the AFLP analysis procedure is complicated and costly. All these restrict the application for large-scale screening in breeding programs. Thus, it is necessary to convert AFLP markers into convenient and inexpensive Polymerase Chain Reaction (PCR)-based markers such as SCAR (Sequence Characterized Amplified Region) markers for MAS (marker-associated selection).

We report here the results of a search for sex markers using the AFLP technique to find differential DNA expression between mature male and female *Eucommia ulmoides* Oliv. The objective was to identify AFLP markers for sex and convert them to SCAR markers for early confirmation of the sex of plants and for the identification of genes related to sex distinction.

## 2. Experimental Section

### 2.1. Plant Material and Genomic DNA Isolation

Young leaves of 30 male and 30 female individual confirmed *Eucommia ulmoides* Oliv. trees were collected at Northwest A and F University, Yangling, Shaanxi province, and in Lingbao city, Henan province. The leaves from the 60 trees were divided into two groups. Ten males and 10 females were used in the first group for constructing the gene pool; the second group, including the other 40 male and female individuals, was used to verify the markers linked to sex.

Genomic DNA was isolated from the leaves from each individual tree according to the CTAB method with minor modifications [27]. The extracted DNA was diluted to a final concentration of 200 ng/μL. To screen AFLP markers linked to sex, equivalent amounts of DNA from the leaves of 10 male individuals were pooled to construct male bulk NO.1 (BM) and 10 female were pooled to make NO.2 (BF). All genomic DNA was kept frozen at −20 °C for digestion reaction with the AFLP technique.

### 2.2. AFLP Assay

Genomic DNA (500 ng) from the two bulks and 60 individuals was digested for 6 h at 37 °C with 10 U EcoRI (New England BioLabs), 10 U MseI (New England BioLabs), 2.5 μL NEBuffer 4, 0.25 μL 100 × BSA, in a 25 μL reaction volume adjusted with distilled water. In the same tube, 10 μL ligation mixture was added consisting of 1 μL EcoRI adapter (5 pmol/μL), 1 μL MseI adapter (50 pmol/μL), 3.5 μL T4 DNA ligase buffer, 400 U T4 DNA ligase (New England BioLabs) and 3.5 μL distilled water. The reaction was incubated for 3 h at 16 °C and stopped by heating at 65 °C for 10 min. The ligated DNA was stored at −20 °C until pre-amplification.

For pre-amplification, a mix was prepared composed of: 1 μL of ligated DNA, 2.5 μL 10 × Taq DNA polymerase buffer, 0.5 μL of 2 mM dNTPs (Sangon), 0.6 μL EcoRI pre-amplification primer (50 ng/μL), 0.6 μL MseI pre-amplification primer (50 ng/μL), 1.5 μL 25 mM MgCl2, 2.5 U Taq DNA polymerase (Fermentas) and distilled water to a volume of 25 μL. The following cycling parameters were used for pre-amplification: 30 cycles of 30 s at 94 °C, 60 s at 56 °C, and 60 s at 72 °C, followed by 5 min at 72 °C for final extension. The PCR products were stored at −20 °C until selective amplification.

The components for each 25 μL amplification reaction were: 1 μL diluted (1:30) pre-amplified products, 2.5 μL 10 × Taq DNA polymerase buffer, 0.5 μL of 2 mM dNTPs (Sangon), 1 μL of EcoRI + 3 primer (50 ng/μL), 1 μL MseI + 3 primer (50 ng/μL), 1.5 μL 25 mM MgCl2, 2.5 U Taq DNA polymerase (Fermentas) and distilled water. The AFLP products were amplified using touchdown PCR. An initial denaturation consisted of 1 cycle of 30 s at 94 °C, 30 s at 65 °C, and 60 s at 72 °C. The annealing temperature was then lowered by 0.7 °C per cycle during the first 13 cycles, and then 23 cycles were performed at 94 °C for 30 s, 56 °C for 30 s, and 72 °C for 60 s. Finally, selective amplification products were separated in 6% denaturing polyacrylamide gel and visualized by silver nitrate staining.

### 2.3. AFLP Band Isolation and Sequencing

The sex-specific fragments were excised from PAGE and extracted using the Long Range Gel Extraction Kit (CWBio). 1 μL of extraction product was used as template or selective amplification with the same primer combination in the 25 μL PCR reaction mix (as used for first round AFLP amplification, above). The PCR reaction products were separated by electrophoresis in 1.5% agarose gels (Invitrogen). DNA bands were excised from the gel and purified with UNIQ-10 EZ Spin Column DNA Gel Extraction Kit (Sangon), and the DNA was cloned into the plasmid vector pGEM-T (Promega). Colonies containing target fragments were sequenced by BeiJing BGI Co. Ltd.

### 2.4. Conversion of AFLP Marker into SCAR Marker

Primers were designed from the sequences of AFLP markers or extended AFLP markers using the software Primer 3. These primers were used to amplify genomic DNA of 30 male and 30 female individuals.

The SCAR reaction was carried out in a 25 μL mixture, containing 1 μL template DNA, 2.5 U Taq DNA polymerase, 1.5 μL 25 mM MgCl2, 0.5 μL of 2 mM dNTPs and 5 pmol each of forward and reverse primers as well as 2.5 μL 10 × PCR reaction buffer. The annealing temperature of the SCAR primers was optimized first, using the following cycling parameters: one cycle of 2 min at 94 °C; 30 cycles of 30 s at 94 °C, 30 s at an annealing temperature, and 80 s at 72 °C. Then 5 μL PCR products from each sample were examined in a 1.5% agarose gel to confirm whether the SCAR primers were converted successfully.

## 3. Results and Discussion

**Figure 1:**
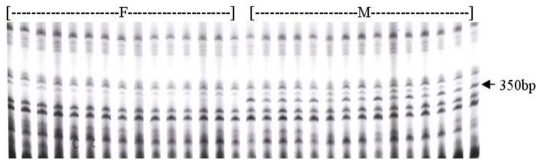
Banding pattern of the male-specific AFLP marker (arrowhead) detected with the primer combination E-ACA/M-CTT. A 350 bp band is present in the males (M) but not the females (F).

In the AFLP assay combined with BSA, 64 AFLP primer combinations were used to identify putative markers in two DNA bulks of male and female individuals. Of these, nine primer combinations revealed polymorphism between two DNAs bulks. To confirm that the markers were linked to sex, 20 male and 20 female individuals were tested independently. The result shows that only primer combination E-ACA/M-CTT produced a 350 bp male-specific marker from all male samples that was absent from all female individuals (Figure 1).

**Figure 2:**
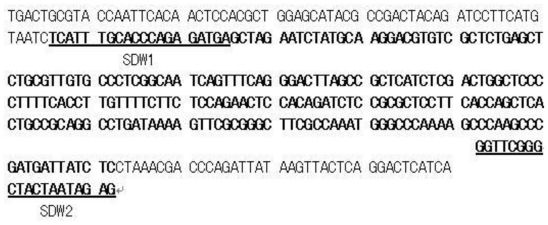
The sequence of the male-specific marker. SDW1 and SDW2 were designed forward and reverse primers (underlined) for the SCAR marker. The bold section designates the sequence of the SCAR marker.

**Figure 3:**
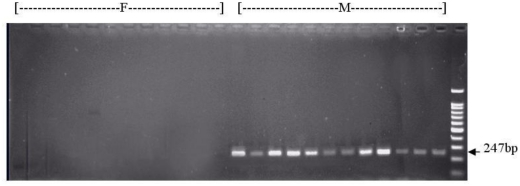
The SCAR marker linked to sex in *Eucommia ulmoides* Oliv. analysis with the primer combination SDW1 and SWD2 of individual plants obtained from the male plant (arrow). A 247 bp band is present in the males (M) but not in the females (F).

The sequence of the cloned male-specific fragment was determined. Based on the sequence, primers SDW1 and SDW2 were designed (Figure 2). The optimized annealing temperature of the SCAR primers was 65 °C. At this annealing temperature, a 247 bp SCAR marker was obtained. The agarose gel in Figure 3 shows that the marker was observed in all the male plants (M) but none of the female plants (F). The sequence of the 247 bp SCAR marker was determined, and it is completely coincided with the corresponding sequence of the AFLP maker. It is clear that the male-specific AFLP marker was successfully converted into a SCAR marker.

A search for the sequence in the databases of GENBANK showed that there is no homologous sequence in other species. This result may be expected since AFLP detect random genomic variation, most of which will be non-coding DNA [21].

We have shown that the combined strategy using bulked segregant analysis and AFLP is an efficient method for identifying sex specific markers in *Eucommia ulmoides* Oliv. In this paper, a total 64 pairs of AFLP primer combinations were screened, but only one male-specific marker was indentified. To our knowledge, this is the first report of a sex-specific AFLP marker in *Eucommia ulmoides* Oliv.

AFLP and RAPD (Random amplification polymorphic DNA) are frequently used molecular marker techniques to search for sex markers in dioecious plants [28]. These two molecular marker techniques have different advantages and shortcomings. Although the most simple and convenient technique used in practice, RAPD has low repeatability and stability [29–32]. AFLP can produce large number of candidate markers in a single experiment and it is highly reproducible [33]. However, AFLP markers are relatively costly and the method is technologically demanding, and this limits its application in breeding programs [34]. Thus, we converted an AFLP marker into a convenient and inexpensive PCR-based marker SCAR. A SCAR marker is a genomic DNA fragment at a single genetically defined locus that is identified by PCR amplification using a pair of specific primers. We derived the SCAR marker by cloning and sequencing the two ends of the amplified products of AFLP markers. The sequence was used to design pairs of primers that resulted in the reproducible amplification of single loci when high annealing temperatures were used. SCAR markers are advantageous over AFLP markers as they detect only a single locus, their amplification is less sensitive to reaction conditions, and they can be put in practice expediently [35].

There are a number of potential uses for sex specific markers, both in applied and basic research. In applied research for *Eucommia ulmoides* Oliv., plants require seven to eight years to reach reproductive maturity before the physical identification of sex of plants is possible, sex identification by molecular marker allows for early confirmation of sex without waiting for sexual maturity. Moreover, since there are distinct different practical uses between the two sexes of *Eucommia ulmoides* Oliv., early sex identification could be economically important for the breeding of this species. In basic research, isolation and characterization of the sex markers from mature *Eucommia ulmoides* Oliv. could provide valuable data for understanding the mechanisms of sex determination.

## 4. Conclusions

Here we present a male-specific AFLP and SCAR marker, which was stable in samples from two locations (Henan and Shaanxi province). This indicates that the male-specific marker linked to sex in *Eucommia ulmoides* Oliv. was not affected by environmental conditions. Thus, the male-specific marker could be recommended as a reliable genetic sex linked marker and hence used to detect the sexuality of *Eucommia ulmoides* Oliv. This result offers a rapid and simple method to determine the sex of *Eucommia ulmoides* Oliv. seedlings at an early stage, thereby saving time and economic resources when cultivating this species.
